# Effect of L-alanine exposure during early life stage on olfactory development, growth and survival in age-0 lake sturgeon *Acipenser fulvescens*

**DOI:** 10.1093/conphys/coae084

**Published:** 2024-12-17

**Authors:** Tyler Edwards, Ian A Bouyoucos, Caleb T Hasler, Mark Fry, W Gary Anderson

**Affiliations:** Department of Biological Sciences, University of Manitoba, 50 Sifton Rd, R3T 2M5 Winnipeg, Manitoba, Canada; Department of Biological Sciences, University of Manitoba, 50 Sifton Rd, R3T 2M5 Winnipeg, Manitoba, Canada; Department of Biology, University of Winnipeg, 515 Portage Ave, R3B 2E9, Winnipeg Manitoba, Canada; Department of Biological Sciences, University of Manitoba, 50 Sifton Rd, R3T 2M5 Winnipeg, Manitoba, Canada; Department of Biological Sciences, University of Manitoba, 50 Sifton Rd, R3T 2M5 Winnipeg, Manitoba, Canada

**Keywords:** Conservation aquaculture, developmental plasticity, foraging behaviour, hatchery, mRNA transcript abundance electro-olfactogram

## Abstract

Environmental factors play an important role in phenotypic development of fishes, which has implications for hatchery-reared fishes that are released into the wild where natural cues are present. There is interest in examining how early exposure to dietary odourants can affect development of olfaction. The aim of our study was to use behavioural, molecular and electro-physiological techniques to evaluate how introduction of the amino acid L-alanine to the rearing environment might influence the development of olfactory perception of dietary cues, growth and survival in lake sturgeon (*Acipenser fulvescens*), a species of conservation concern. We hypothesized that exposure to amino acids would influence the onset of feeding during dietary transitions from endogenous to exogenous feeding and predicted that the introduction of L-alanine during early development would promote growth and survival of age-0 lake sturgeon. Additionally, we hypothesized that olfaction in lake sturgeon is a developmentally plastic trait, predicting that the addition of L-alanine prior to exogenous feeding would influence mRNA transcript abundance of genes associated with detection of dietary cues. Our approach was to add L-alanine daily from 17 to 20 days post-fertilization (DPF) before the onset of exogenous feeding. We sampled individuals at 17, 21, 26, 31, 50, 65 and 80 DPF. Additionally, olfactory sensitivity to L-alanine was tested at ~1 year via electro-olfactogram (EOG). We observed no significant differences in mortality or EOG response between L-alanine and control treatments; however, significant differences were observed in morphometrics, behaviour and mRNA transcript abundance of all genes throughout development. Our results indicated the olfactory system exhibited developmental plasticity in response to L-alanine treatment until 50–65 DPF, suggesting that environmental odourants may influence early development of key olfactory processes. Our data could inform practises at conservation hatcheries that are used as part of enhancement programmes for lake sturgeon.

## Introduction

Lake sturgeon *Acipenser fulvescens* are a slow-maturing freshwater fish found in three major drainage basins in North America: Mississippi River, North American Great Lakes and Hudson Bay (Peterson *et al.,* 2007; Bruch *et al.,* 2016). Although once plentiful, populations in their natural range have been significantly depleted primarily due to habitat loss and overfishing in the late 1800s and early 1900s (Hay and Whelan, 1997; Peterson *et al.,* 2007). Since these declines, there have been further human-induced changes to their environment, such as fragmentation by hydroelectric dams (Mcdougall *et al.,* 2014, Osborne *et al.,* 2020). Ultimately, when such anthropogenic-induced changes are combined with slow maturation of lake sturgeon, population growth is challenged and conservation measures are often needed.

Conservation hatcheries have been part of conservation strategies for many fish species and are almost ubiquitously employed across the natural range of lake sturgeon where juveniles are raised from gametes collected from native, wild-caught broodstock. Early life stages are grown in the hatchery environment, which dramatically increase survival throughout the first year of life (Anderson *et al.,* 2022). Once past their most vulnerable stages fish are released back into their native environment to enhance the existing population. Whilst conservation hatcheries have been used continuously to improve many fish stocks, the process can result in lower performing phenotypes (Álvarez and Nicieza, 2003; Orlov *et al.,* 2006; Blanchet *et al.,* 2008; Roberts *et al.,* 2011; Saikkonen *et al.,* 2011; Stringwell *et al.,* 2014; Duda and Kapusta, 2019).

An important phenotype seldomly assessed in conservation strategies is development of the olfactory sensory system. With respect to the detection of food and resulting behaviour, it is relatively common practise for food, and by default the associated odour, not to be introduced until absorption of the yolk-sac is nearly complete (Gisbert and Williot, 2002, 1997). In Siberian sturgeon *Acipenser baerii* yolk-sac absorption is marked by cessation of schooling behaviour (Gisbert and Williot, 2002, 1997). Moreover, the timing of food introduction varies by water temperature and species, generally occurring between 18 and 26 days post-fertilization (DPF) as reviewed by Anderson *et al.* (2022). Interestingly, if food is not provided before yolk-sac absorption is complete, larvae may fail to begin feeding, which leads to mortality (Gisbert and Williot, 1997; Chai *et al.,* 2014). Nevertheless, following yolk-sac absorption fish are typically fed a diet of zooplankton (e.g. *Artemia*) prior to transition to a larger invertebrate or commercial pelleted diet (Buddington and Christofferson, 1985; Gisbert and Williot, 1997; Boglione *et al.,* 1999; Gessner *et al.,* 2009; Earhart *et al.,* 2020; Yoon *et al.,* 2022). This approach promotes growth and development of the fish prior to stocking in the wild; however, there remains limited success in recruitment of lake sturgeon from conservation hatchery facilities (Osborne *et al.,* 2020), particularly when released as fall fingerlings (Mcdougall *et al.,* 2014). Olfaction is considered the primary sensory system for detecting food in Chinese sturgeon *Acipenser sinensis* (Liang *et al.,* 2011), suggesting it may also play a role in recruitment challenges faced by stocked lake sturgeon. The disparity between odourants in hatcheries and those in the wild may impede the ability of hatchery-reared fish to recognize wild odourants, impacting their survival. Introducing wild odourants to the hatchery environment could enhance individuals’ olfactory recognition of said odourants, potentially improving their recruitment success.

Environmental factors at early life stages play a large role in phenotypic development that may persist into adulthood (Piersma and Drent, 2003; Johnsson *et al.,* 2014; Stringwell *et al.,* 2014). In fishes, environmental factors include interactions with conspecifics, encountering predators, habitat complexity, food availability, natural prey and possibly odour cues (Johnsson *et al.,* 2014; Anderson *et al.,* 2022). The presence or absence of any of these factors may influence the phenotypic development of an individual. Changes in phenotypes can be advantageous modifying morphology, physiology and behaviour to suit one’s environment (Beldade *et al.,* 2011; Teletchea and Fontaine, 2014). In the artificial environment of conservation hatcheries, many of these environmental factors are missing leading to the development of lower performance phenotypes prior to release to the wild. For instance, hatchery-reared brown trout, *Salmo trutta*, showed increased naivety to predators once released (Álvarez and Nicieza, 2003; Stringwell *et al.,* 2014,), and Atlantic salmon, *Salmo salar*, had challenges recognizing natural prey (Orlov *et al.,* 2006). In the case of lake sturgeon, hatchery-reared individuals demonstrated a decreasing response to alarm cues with age (Wishingrad *et al.,* 2014) and showed preference to their hatchery diet over a wild-type diet in a behavioural arena (Edwards *et al.,* 2024). Still very little is known about how these performance metrics in a hatchery setting may translate to success in the wild, despite their potential influence on low recruitment success in lake sturgeon (Osborne *et al.,* 2020).

In studied fishes, odourants are detected at the apical surface of the olfactory epithelium by receptors of the olfactory sensory neurons (OSNs) (Kasumyan, 2004; Hara, 2011). In sturgeons, the olfactory epithelium contains three types of OSNs: ciliated, microvillous and crypt (Zeiske *et al.,* 2003; Kasumyan, 2004). Located on the cilia and microvilli of OSNs are four main types of receptors, each part of the superfamily of G protein-coupled receptors (GPCRs). These include olfactory receptors (ORs), trace-amine-associated receptors (TAARs) and two types of vomeronasal receptors, V1R and V2R, which may bind specific and/or general odourants (Hara, 2011; Olivares and Schmachtenberg, 2019). Importantly, there are numerous isoforms of each receptor type with a total of 305 receptors described in zebrafish *Danio rerio (*Gerlach *et al.,* 2019*).* In teleosts, ORs and TAARs are the dominant receptor type expressed on ciliated OSNs, and vomeronasal receptor (V2R) are the dominant type expressed on microvillous OSNs (Olivares and Schmachtenberg, 2019). The ciliated OSNs and associated receptors are referred to as generalist as they bind a number of odourants including amines, amino acids, prostaglandins and bile acids, resulting in different behaviours such as conspecific communication and predator avoidance (Sato and Suzuki, 2001; Hubbard *et al.,* 2003; Liberles and Buck, 2006; Gerlach *et al.,* 2019). In comparison, vomeronasal receptors located on microvillous OSNs are considered specialist typically linked with odourants associated with food (Sato and Suzuki, 2001; Korsching, 2009); specifically, these receptors are thought to have evolved to bind a diverse array of amino acids (Alioto and Ngai, 2006). Amino acids are widely associated with olfactory odourants most notably for feeding behaviour in teleosts and sturgeon. In teleosts and sturgeon, the most efficacious amino acids producing strong neurophysiological and behavioural responses have been characterized as short, simple and straight-chained, such as L-alanine and glycine (Laberge and Hara, 2004; Hara, 2011; Shamushaki *et al.,* 2011; Edwards *et al.,* 2024).

The aim of our study was to understand biological responses to the introduction of L-alanine in the early rearing environment of lake sturgeon and we used behavioural, molecular and electro-physiological techniques to evaluate the development of olfactory perception of dietary cues, growth and survival in age-0 lake sturgeon. We hypothesized that L-alanine exposure may influence the onset of feeding during dietary transitions from endogenous to exogenous feeding and that olfaction in fishes is a developmentally plastic trait. We predicted that introduction of L-alanine during early life history would have a positive effect during dietary transitions and therefore promote growth and survival of age-0 lake sturgeon. Associated with the positive benefit of L-alanine exposure and developmental plasticity, we predicted that addition of L-alanine prior to exogenous feeding would increase foraging activity in the behavioural arena, increase mRNA transcript abundance of key genes associated with detection of dietary cues and increase electro-physiological sensitivity to dietary cues later in life.

## Materials and Methods

### Animal husbandry

**Figure 1:**
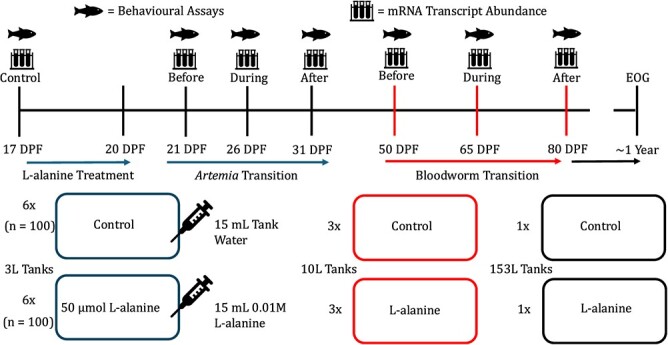
A visual depiction of the experimental design, including timeline, treatments and sampling points for larval lake sturgeon *A. fulvescens*. During the endogenous feeding stage from 17 to 20 DPF, 15 ml of 0.01 M L-alanine, to give a final concentration of 50 μmol, was added to six treatment tanks and the equivalent amount of untreated aquarium water was added to the remaining six tanks as a control and left stagnant for 1 h. At 50 DPF, 12 tanks were condensed into six larger (10 l) tanks (three per treatment) with similar fish densities. Following bloodworm transition ~103 DPF, fish were transferred to larger 153-l tanks to be on-grown (colour of arrows corresponds to size of aquaria Blue = 17–31 DPF 3-l tanks, Red = 50–103 DPF 10-l tanks and Black = 103 DPF–1 year 153-l tanks). Larvae were sampled at 17, 21, 26, 31, 50, 65 and 80 DPF for behavioural experiments and were euthanized with an overdose of MS-222 (250 mg•l^−1^). Both body mass to the nearest 0.001 g, and total length to the nearest millimetre were recorded at each time point for each treatment for all fish used in the behavioural trials. A subset of the fish used in the behavioural trials was used for molecular sampling. Additionally, when fish were ~1 year old, they were tested under EOG to determine if there was any effect of L-alanine treatment.

Mature lake sturgeon were captured in 2021 from the Winnipeg River (Manitoba, Canada; Manitoba scientific collectors permit 22 554 910), downstream of Pointe du Bois generating station in Slave Falls reservoir (50.3008° N, 95.5514° W). Gametes were collected and ~15 ml of eggs from one female were fertilized with 200 μl of milt from two males (Mims *et al.,* 2002). Embryos were placed in McDonald tumbling jars and reared at 12°C until hatch ~12–13 DPF. Once all larvae were hatched, 100 free-swimming larvae were transferred to each treatment tank (Fig. 1) and the temperature was gradually increased from 12 to 15.5°C over 5 days in all tanks. Fish were on grown in flow-through aquaria fed by dechlorinated City of Winnipeg tap water at a temperature of 15.5 ± 1°C. Larvae were fed a diet of *artemia* (*ad libitum*) three times a day at 8 am, noon and 8 pm from 21 to 49 DPF and slowly transitioned to a diet of bloodworm between 50 and 80 DPF with a 10% increase in bloodworm occurring every 3 days. Larvae were allowed to feed for ~30 min or until cessation of feeding behaviour, and any uneaten food was removed by siphon. Additionally, at each feeding time we removed and recorded mortalities.

### Experimental design

During the endogenous feeding stage from 17 to 20 DPF, flow to each tank was turned off at noon for amino acid treatment. We added 15 ml of 0.01 M L-alanine to six treatment tanks, resulting in a final concentration of 50 μmol, and an equal volume of untreated aquarium water was added to the remaining six tanks as a control (Fig. 1). The tanks were left stagnant, but with constant aeration, for 1 h before water flow was turned back on. Importantly, the final concentration of L-alanine in the treatment tanks was slightly above the electro-olfactogram (EOG) detection threshold observed in age-1+ lake sturgeon (1 × 10^−6^ M; Edwards *et al.,* 2024). Larvae were sampled at seven time points from 17 to 80 DPF for behavioural and molecular end points (Fig. 1). Notably, the 17- and 21-DPF sampling points represent the period before and after L-alanine treatment, prior to any introduction of *artemia*. During sampling, six larvae were sampled from each replicate tank from 17 to 36 DPF and 12 larvae were sampled from each replicate tank from 50 to 80 DPF (Fig. 1). Larvae were haphazardly selected by dip net to be used in behavioural trials then euthanized with an overdose of tricaine mesylate (MS-222, 250 mg•l^−1^) equally buffered with NaHCO_3_^−^. Body mass to the nearest 0.001 g (Explorer Pro, Ohaus New Jersey, USA) and total length to the nearest millimetre were recorded at each time point for each treatment.

### Behavioural experiments

Behavioural responses to diet cues were assessed at 17, 21, 26, 31, 50, 65 and 80 DPF to encompass all periods including before the L-alanine treatment as well as before, during and after the *artemia* and bloodworm dietary transition (Fig. 1). As lake sturgeon grew the behavioural arenas increased in size from a 90-mm-diameter petri dish (50 ml) at 17 DPF (*n* = 36, length, mean ± SD = 14.7 ± 0.5 mm), 21 DPF (*n* = 36, length = 17.8 ± 0.3 mm) and 26 DPF (*n* = 36, length 20.7 ± 0.4 mm) to a rectangular arena measuring 75 mm L × 100 mm W × 35 mm H (200 ml) at 31 DPF (*n* = 36, length 22.8 ± 0.4 mm) and 50 DPF (*n* = 36, length 36.8 ± 1.5 mm), and a larger arena measuring 150 mm L × 100 mm W × 35 mm (400 ml) at 65 (*n* = 36, length 45.2 ± 2.4 mm) and 80 DPF (*n* = 36, length 48.5 ± 4.4 mm). This was done to maintain the arena-to-fish ratio consistent with our previous study, where we observed significant behavioural differences in juvenile lake sturgeon (Edwards *et al.,* 2024). All arenas were white in colour. Curtains were placed around the outside of the arena to prevent external disturbance, whilst additional light sources were installed above the arena to provide sufficient light and eliminate glare from the surface of the water. At the beginning of a trial arenas were pre-filled with untreated aquarium water. Six larvae were haphazardly selected by dip net and individually placed in an arena to recover for 2 min prior to the start of the trial (Gouraguine *et al.,* 2017; Wassink *et al.,* 2020). At the beginning of the 5-min exposure either a diet cue (*artemia* or bloodworm) or a blank cue (untreated tank water) was injected into the middle of the arena. For each trial, only one treatment at a time was tested exposing three arenas to the blank and three to the diet cue. *Artemia* was delivered as the diet cue for trials between 17 and 31 DPF and bloodworm was delivered for trials between 50 and 80 DPF based on diet type at time of trial. The diet cues for the behavioural trial were made fresh daily by adding 0.5 g of the diet type to 1 l of untreated aquaria water and stirred vigorously for 30 min. The diet solution was then filtered by gravity through filter paper (114 wet strengthened, Whatmans, Fisher Scientific). The same volume of cue, 0.2% of total volume, was added to the arenas for each trial to maintain a consistent concentration of cue during each life stage. Equivalent volumes of untreated aquarium water were added in the same fashion at all life stages as a blank. Cues were randomly distributed across arenas for each trial and a total of 18 replicates were conducted for each cue at each time point for each treatment. Additionally, after each trial the arenas were scrubbed with Oxivir (Diversay, South Carolina, USA) and rinsed thoroughly with untreated aquaria water. Behaviour was recorded at 30 fps using a video camera (Sony DCR-SR68, Tokyo, Japan) and analysed using tracking software (Noldus EthoVision XT, Version 15, Wageningen, Netherlands) and output was generated every 0.33 s for the 7-min recording. Videos were trimmed and only the 5-min exposure period was analysed. Distance travelled (cm) of the individual larvae were measured and standardized to body length (BL).

**Table 1:** Standard curve efficiency for qPCR primers in two tissues in 17–36 DPF (whole body) and 50–80 DPF (head only) lake sturgeon *A. fulvescens*

Gene	F-Primer	R-Primer	Whole body (%)	Head (%)
*v2r 1-like*	GCGTACTTGAGTACCAGGGG	GCCAGCAAGCCAAAACTTGA	105	105.2
*v2r 26-like*	CCTCTGAATGGTCCTGTATGTT	GCCATTGACATGCCTATTGTTT	103.7	95.5
*or 1-like*	AGTACAGCGGTAATCCCAAATC	TGCCAAGAGCACAAGAGAAA	106.8	95.5
*taar 1-like*	CTGTCAGAGAGAGGGTGAGATA	CTGTTTGTGGAAACCTGTTTGT	101	105.2
*ef1a*	TGGCATCACCATTGACATCT	AGCTGCTTCACACCCAGAGT	98.7	103.5
*rsp6*	CTGGCTGGATTCTGATTTGGATG	ATCTGATTATGCCAAGCTGCTG	100	102.4

### mRNA transcript abundance

A subset of the fish used in behavioural assays exposed to the blank cue at 17, 21, 26, 31, 50, 65 and 80 DPF from each treatment were used in assessing mRNA transcript abundance of target genes. A total of seven individuals per time point and treatment were haphazardly selected immediately following the behavioural assays from randomized tanks. Following euthanasia larvae were placed in a 1.7-ml snap cap tube with 0.5–1 ml RNAlater (Invitrogen; Thermo Fisher Scientific Waltham, USA), stored at 4°C for 24 h and then at −80°C prior to RNA extraction and molecular analysis. Importantly, the size of individuals constrained our ability to target the olfactory tissue during early development. As a consequence, we measured whole-body transcript abundance of target genes for samples between 17 and 31 DPF and transcript abundance of target genes from the head region (i.e. tissue anterior to the gill slits) in fish sampled between 50 and 80 DPF.

Samples were homogenized individually in lysis buffer from Purelink RNA mini kit (Invitrogen, CA, USA) using a mini bead mill homogenizer (VWR) for 60 s. Total RNA was extracted following the manufacturer’s instructions. RNA yields were quantified spectrophotometrically using a nano-drop (Thermo-Scientific nanodrop 2000c spectrophotometer) and quality was assessed by 1% agarose gel with ethidium-bromide and visualized band integrity under UV light.

RNA samples were then diluted using RNase free water to an approximate concentration of 0.2 μg•μl^−1^ and any residual DNA was digested with the addition of Deoxyribonuclease I Amplification Grade (cat. NO. 18068–015; Invitrogen; Life Technologies) following the manufacturer’s instructions. Complementary DNA synthesis was completed using an iScript cDNA synthesis kit (Cat. NO. 1708891, Bio-Rad) following the manufacturer’s instructions. The samples were immediately diluted to a total volume of 40 μl using RNA-free water and stored at −20°C prior to subsequent analysis.

We conducted real-time quantitative polymerase chain reaction (RT-qPCR) on two microvillous OSN related receptors (*v2r 1-like, v2r 26-like*) and two ciliated related receptors (*taar 1-like* and *or 1-like*) using a 2-fold dilution of cDNA template for all genes of interest. Each reaction used Bio-Rad SYBR green master mix (Bio-Rad Laboratories, CA, USA) with total volume of reaction being 12 μl. All genes were found in the NCBI database in sterlet sturgeon *Acipenser ruthenus* and the open reading frame was blasted using NCBI Blast P in paddlefish *Polyodon spathula* to assess their alignment from which the most conserved region was used to design primers. Initial presences of genes of interest were assessed by PCR using Bio-rad T100 thermal cycler (Bio-Rad Laboratories, CA, USA) in age-1 lake sturgeon olfactory epithelium (data not shown). Following PCR, we assessed qPCR primer efficiency by using a 1:2 dilution standard curve (Table 1–2). All reactions were conducted using a Bio-Rad CFX Connect qPCR machine (Bio-Rad Laboratories, CA, USA) in 96-well plates and under identical conditions: 2 min at 95°C, 40 cycles of 15 s at 95°C, 30 s at 58°C and 30 s at 72°C. Melt curves were determined by denaturation for 15 s at 95°C, a decrease for 1 min down to 60°C, followed by a gradual increase of 0.075°C s^−1^ to 95°C. qPCR amplification data was normalized using the Norma-gene normalization method described by Heckmann *et al.* (2011). The Norma-gene normalization method is optimized when there are five or more genes included in the analysis so genes *rsp6* and *ef1α* were used in the analysis although data is not reported. The *rsp6* and *ef1α* primer sequences used were based on previous work on larval lake sturgeon from our lab (Earhart *et al.,* 2020).

### EOG recording

The EOG experiments were performed as previously described by (Edwards *et al.,* 2024). The EOG experiments were conducted using fish at ~1 year post-fertilization to assess if exposure to L-alanine during early development influenced the responsiveness of individuals to a standard set of environmental cues. The EOG responses of individuals to a total of three environmental cues were examined. L-alanine was used as a standard food cue and a possible imprinting amino acid presumably acting through microvillus OSNs; taurocholic acid (TCA) was used as a standard stimulant for presumably ciliated OSNs; and a mixed cue of filtered commercial trout pellet diet (EWOS 5 mm), which represented the diet of the fish at time of sampling. Odourant concentrations for L-alanine and TCA followed previous literature with concentrations of 10^−3^ M and 10^−4^ M, respectively (Sakamoto *et al.,* 2016; Edwards *et al.,* 2024). The commercial trout pellet cue was prepared as mentioned above except all odourants and cues were made up in artificial fresh water (AFW mentioned below); as well, AFW was used as a vehicle control to normalize the data. AFW was prepared in Milli-Q water, with a final ionic concentration of (in mM) 0.4 Na^+^; 0.4 Ca^+^; 0.04 K^+^; 0.84 Cl^−^; and 0.4 HCO_3_^−^ (Mcintyre *et al.,* 2008). Each fish was exposed to each odourant at least three times in a randomized order. To normalize the data the EOG response to the vehicle control (AFW) was subtracted from the EOG response of each cue and that was then normalized to 10^−3^ M L-alanine, which was sampled in triplicate prior to first randomized sequence so that all cues for a single fish would be normalized to the baseline of initial responses of 10^−3^ M L-alanine.

**Figure 2:**
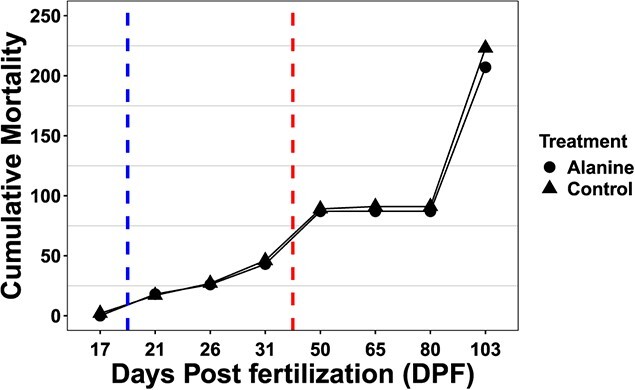
Cumulative mortality of age-0 lake sturgeon *A. fulvescens*. Blue dotted line signifies the start of the *artemia* transition, and the red dotted line signifies the start of the bloodworm transition. Each point represents cumulative mortality for each treatment (L-alanine or Control).

**Figure 3:**
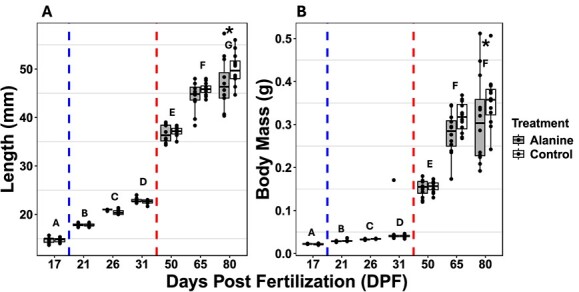
Mean length (mm) (A) and body mass (g) (B) of three larval lake sturgeon (*n* = 12) *A. fulvesens*. Blue dotted line signifies the start of the *artemia* transition, and the red dotted line signifies the start of the bloodworm transition. Similar letters above the bars denote statistically similar groups LME (*P* < 0.05). Asterisks denote statistical differences between treatment groups LME (*P* < 0.05). Data are expressed as median Q2 = dark line in the box, with 25th percentile Q1 = low line, 75th percentile Q3 = upper line (which make up the interquartile range, IQR), whiskers represent the minimum (minimum value in the data Q1 – 1.5*IQR) and maximum (maximum value in the data, Q3 + 1.5*IQR).

## Statistical Analysis

Analyses were completed using R (version 1.4.1106; R Core Team, 2021). Assumptions of normality and homogeneity of variance were visually assessed using the check_model() function from the package ‘performance’ (Zuur *et al.,* 2010), which produced quantile–quantile plots of residuals and plotted dependant variables against residuals of linear mixed effect (LME) models and analyses of variance (ANOVAs). If assumptions were not met, the data was natural-log or rank transformed. The LME models used package ‘lme4’ (Bates *et al.,* 2015); *P*-values and degrees of freedom for LME were estimated using ‘car’ package (Fox, 2023), with degrees of freedom derived from Wald chi-square tests. *Post hoc P*-values were established using the ‘emmeans’ package (Lenth *et al.,* 2021) with a Bonferroni *P*-value correction. Prior to Bonferroni correction, the type I error rate for this analysis was α = 0.05.

Differences in mortality between treatments were analysed via Cox proportional hazards model using the ‘survival’ and ‘survminer’ packages (Therneau, 2015; Kassambara *et al.,* 2019; Bugg *et al.,* 2020). Assumptions for Cox proportional hazard model were assessed using the cox.zph() function included in the ‘survival’ package, ensuring that residuals were independent of time.

Morphometrics including mass (g) and length (mm) during development were analysed using LME models with DPF, treatment and their interactions included in the models as fixed effects, whilst tank ID was included as a random effect. Similarly, in the behaviour experiment LME models were used to analyse total distance (BL); following cue injection with treatment, cue and their interactions included in the model as fixed effects and tank ID as a random effect. We used one or two-way ANOVAs to analyse mRNA transcript abundance of olfactory genes with DPF, treatment and their interactions included in the model as fixed effects. The mRNA transcript abundance data was partitioned in accordance to points of development, endogenous feeding 17 DPF, artemia diet transition 21–31 DPF and bloodworm transition 50–80 DPF. Finally, EOG responses were analysed using two-way ANOVAs with cue, treatment and their interactions included in the models as fixed effects.

## Results

### Mortality & morphometrics

We observed no significant statistical difference in mortality over the experiment period between L-alanine and control treated fish with a hazard ratio of 0.379 (*P* > 0.05) (Fig. 2). Further, throughout the experimental period we assessed length (mm) and body mass (g) at each time point (17, 21, 26, 31, 50, 65, and 80 DPF). For these measurements, the body mass is the mean mass of three individuals stored in the same 1.7-ml tube. Similarly, the length is the mean length of those same three individuals. Therefore, we condensed 36 individuals into 12 mean points for each time point per treatment. We observed a significant main effect of DPF on length (rank transformed LME; χ^2^ (6) = 5764.358, *P* < 0.001) but also report a significant interaction effect between the treatment and DPF on length (rank transformed LME; χ^2^ (6) = 21.943, *P* = 0.001). *Post hoc* analysis revealed at 80 DPF the mean length of L-alanine treatment was significantly less than control (*P* < 0.01) (Fig. 3a). Further, there was a significant main effect of DPF on body mass (rank transformed LME; χ^2^ (6) = 4327.141, *P* < 0.001). *Post hoc* analysis found no significant difference between 65 and 80 DPF (*P* = 0.189). We also report a significant interaction between treatment and DPF on body mass (g) (rank transformed LME; χ^2^ (6) = 14.169, *P* = 0.023). *Post hoc* analysis revealed at 80 DPF the mean body mass of L-alanine treatment was significantly less than control (*P* < 0.01) (Fig. 3b).

### Behavioural experiment

**Figure 4:**
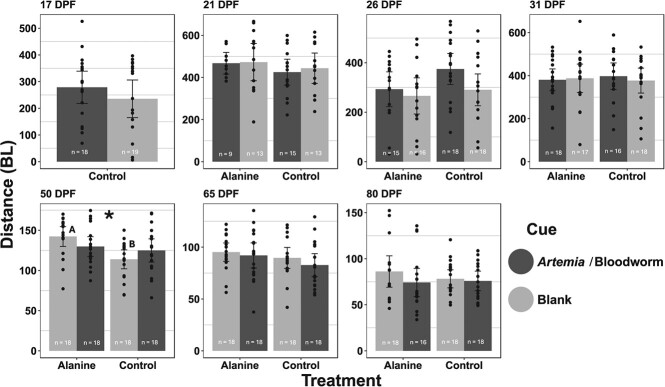
Total distance (BL) of lake sturgeon (*Acipenser fulvescens*) after exposure to appropriate diet cue. Black-shaded bars represent *artemia* (17–31 DPF) or bloodworm (50–80 DPF) and grey-shaded bars = blank (untreated aquaria water). Fish were raised in either L-alanine or control treatment tanks. Similar letters above the bars denote statistically similar groups LME (*P* < 0.05). Asterisks denote statistical differences in treatment LME (*P* < 0.05). Data are expressed as mean ± SEM.

In all behavioural trials, except at 17 DPF prior to treatment with L-alanine where we only compared effect of cue, we compared the foraging activity to the current diet cue (*artemia* or bloodworm) to blank (untreated aquaria water) to assess any differences in total distance (BL) between L-alanine and control treatments. We observed a significant main effect of treatment at 50 DPF for total distance (LME; χ^2^ (1) = 5.757, *P* = 0.016; Fig. 4) where the L-alanine-treated larvae total distance was greater than that of the control (*P* < 0.05). Additionally, we report a significant interaction effect of treatment and cue at 50 DPF for total distance (LME; χ^2^ (1) = 3.8739, *P* = 0.049; Fig. 4). *Post hoc* analysis revealed when L-alanine-treated larvae were exposed to the blank cue their total distance travelled was greater than that of the control (L-alanine mean ± standard error of the mean (SEM) 142 ± 6.48 and control 114 ± 6.48) (*P* < 0.01).

### mRNA transcript abundance

#### v2r 1-like

**Figure 5:**
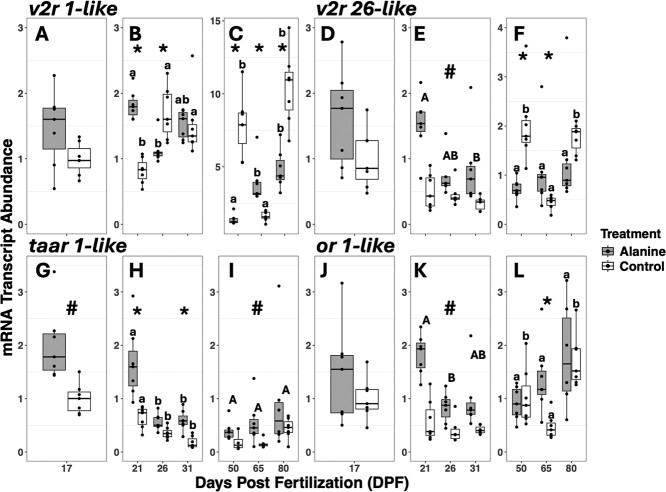
mRNA transcript abundance of *v2r 1-like, v2r 26-like, taar 1-like and or 1-like* in larval lake sturgeon *A. fulvescens*. A, D, G, J = pre-treatment (*n* = 6–7) B, E, H, K = after treatment before, during and after the *artemia* transition (*n* = 6–7) C, F, I, L = after treatment before, during and after the bloodworm transition (*n* = 4–7). Fish were sampled at 17 (pre-treatment), 21, 26, 31, 50, 65 and 80 DPF. Sampled tissues were the whole body (17–31 DPF) and head (50–80 DPF). Treatment represents tanks treated with either L-alanine or control (untreated aquaria water). Similar lower case letters above the bars denote statistical similar groups within treatment two-way ANOVA (*P* < 0.01), asterisks above the bars denote statistical differences between treatments (*P* < 0.01). Similar upper case letters above the bars denote statistical differences in DPF two-way ANOVA (*P* < 0.05), hashtag denotes statistical differences in treatment (*P* < 0.05). Data are expressed as median Q2 = dark line in the box, with 25th percentile Q1 = low line, 75th percentile Q3 = upper line (which make up the IQR), whiskers represent the minimum (minimum value in the data Q1 – 1.5*IQR) and maximum (maximum value in the data, Q3 + 1.5*IQR).

Broadly, mRNA transcript abundance of *v2r 1-like* changed with DPF and treatment following L-alanine exposure with a general increase across time. We observed no significant differences in mRNA transcript abundance of *v2r 1-like* prior to the addition of L-alanine at 17 DPF between the tanks allotted to L-alanine and control treatments (natural-log transformed one-way ANOVA; F_1,12_ = 2.369, *P* = 0.149; Fig. 5a*v2r 1-like*). There was a significant interaction effect between treatment and DPF on mRNA transcript abundance of *v2r 1-like* receptor during the *artemia* transition at 21–31 DPF (natural-log transformed two-way ANOVA; F_2,36_ = 31.811, *P* < 0.001; Fig. 5b*v2r 1-like*). *Post hoc* analysis revealed at 21 DPF the mRNA transcript abundance of L-alanine treatment was significantly higher than control (*P* < 0.01). At 26 DPF, control was significantly higher than L-alanine (*P* < 0.01), but there was no significant difference at 31 DPF (*P* > 0.05). We observed a significant interaction effect between treatment and DPF on mRNA transcript abundance of *v2r 1-like* during the bloodworm transition 50–80 DPF (natural-log transformed two-way ANOVA; F_2,36_ = 88.429, *P* < 0.001; Fig. 5c*v2r 1-like*). *Post hoc* analysis revealed at 50 DPF the mRNA transcript abundance of control was significantly higher than L-alanine treatment (*P* < 0.01), at 65 DPF L-alanine was significantly higher than control (*P* < 0.01) and at 80 DPF the control was significantly higher than L-alanine (*P* < 0.01). The mRNA transcript abundance of *v2r 1-like* in the L-alanine-treated fish also significantly increased from 50 to 80 DPF.

**Figure 6:**
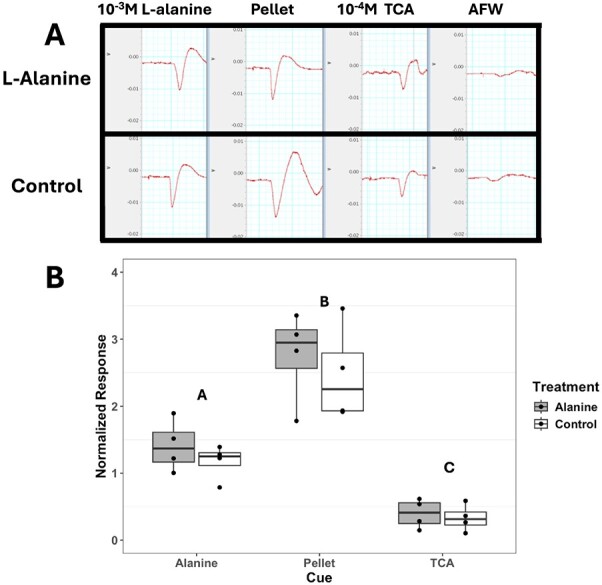
EOG responses of control (*n* = 4) and Alanine-treated (*n* = 4) lake sturgeon *A. fulvescens* to 10^−3^ M L-alanine, pellet cue and 10^−4^ M TCA. A) Representative raw EOG traces of lake sturgeon olfactory epithelia in response. The X-axis represents time with each grid square representing 2 s. The Y-axis represents the resting field potential with each grid square representing 2 mV. B) The normalized response is the normalized amplitude of the mean EOG response per individual. Statistical letters above the bars denote statistically similar groups. Two-way ANOVA (*P* < 0.05). Data are expressed as median Q2 = dark line in the box, with 25th percentile Q1 = low line, 75th percentile Q3 = upper line (which make up the IQR), whiskers represent the minimum (minimum value in the data Q1 – 1.5*IQR) and maximum (maximum value in the data, Q3 + 1.5*IQR).

#### v2r 26-like

Generally, mRNA transcript abundance of *v2r 26-like* changed with DPF and treatment following L-alanine exposure. We observed no significant differences in mRNA transcript abundance of *v2r 26-like* prior to the addition of L-alanine at 17 DPF between the tanks allotted to L-alanine and control treatments (natural-log transformed one-way ANOVA; F_1,12_ = 3.240, *P* = 0.097; Fig. 5d*v2r 26-like*). There was a significant main effect of treatment on mRNA transcript abundance of *v2r 26-like* during the *artemia* transition 21–31 DPF (natural-log transformed two-way ANOVA; F_1,32_ = 36.340, *P* < 0.001; Fig. 5e*v2r 26-like*), with mRNA transcript abundance being higher in L-alanine-treated fish. There was also a main effect of DPF on mRNA transcript abundance (natural-log transformed two-way ANOVA; F_2,32_ = 4.897, *P* = 0.014; Fig. 5e*v2r 26-like*). *Post hoc* analysis revealed at 21 DPF the mRNA transcript abundance of *v2r 26-like* was significantly higher than 31 DPF (*P* < 0.05). At 50–80 DPF during the bloodworm transition there was a significant interaction effect between treatment and DPF on mRNA transcript abundance of *v2r 26-like* (natural-log transformed two-way ANOVA; F_2,35_ = 14.346, *P* < 0.001; Fig. 5f*v2r 26-like*). Specifically, at 50 DPF the mRNA transcript abundance of control treatment was significantly higher than L-alanine (*P* < 0.01), at 65 DPF L-alanine was significantly higher than control (*P* < 0.01) and at 80 DPF there was no difference.

#### taar 1-like

mRNA transcript abundance of *taar 1-like* changed with DPF and treatment following L-alanine exposure with a general decrease across time. We observed significant differences in mRNA transcript abundance of *taar 1-like* prior to the addition of L-alanine at 17 DPF between the tanks allotted to L-alanine and control treatments (natural-log transformed one-way ANOVA; F_1,12_ = 19.68, *P* ≤ 0.001; Fig. 5g*taar 1-like*). *Post hoc* analysis revealed the L-alanine tank mRNA transcript abundance was significantly higher than the control tank (*P* < 0.05). There was a significant interaction effect between treatment and DPF on mRNA transcript abundance of *taar 1-like* during the *artemia* transition at 21–31 DPF (natural-log transformed two-way ANOVA; F_2,36_ = 3.917, *P* = 0.029; Fig. 5h*taar 1-like*). *Post hoc* analysis revealed at 21 and 31 DPF the mRNA transcript abundance of L-alanine treatment was significantly higher than control (*P* < 0.01). There were also significant decreases in mRNA transcript abundance for *taar 1-like* for both treatments during the *artemia* transition. Further, there was a significant main effect of treatment on mRNA transcript abundance of *taar 1-like* during the bloodworm transition at 50–80 DPF (natural-log transformed two-way ANOVA; F_1,31_ = 12.803, *P* < 0.01; Fig. 5i*taar 1-like*), with mRNA transcript abundance being higher in L-alanine-treated fish (*P* < 0.05). There was also a main effect of DPF on mRNA transcript abundance (natural-log transformed two-way ANOVA; F_2,31_ = 4.406, *P* = 0.021; Fig. 5i*taar 1-like*); however, we did not find significant pairwise differences.

#### or 1-like

Lastly, *Or 1-like* mRNA transcript abundance changed with DPF and treatment following L-alanine exposure similar to *v2r 26-like*. We observed no significant differences in mRNA transcript abundance of *or 1-like* prior to the addition of L-alanine at 17 DPF between the tanks allotted to L-alanine and control treatments (natural-log transformed one-way ANOVA; F_1,12_ = 0.909, *P* = 0.359; Fig. 5j *or**1**-like*). There was a significant main effect of treatment on mRNA transcript abundance of *or 1-like* during the artemia transition at 21–31 DPF (natural-log transformed two-way ANOVA; F_1,32_ = 52.4303, *P* = < 0.001; Fig. 5k *or**1**-like*), with mRNA transcript abundance being higher in L-alanine-treated fish compared to the control (*P* < 0.01). There was also a main effect of DPF on mRNA transcript abundance (natural-log transformed two-way ANOVA; F_2,35_ = 7.022, *P* < 0.01; Fig. 5k *or**1**-like*). *Post hoc* analysis revealed at 21 DPF the mRNA transcript abundance of *or 1-like* was significantly higher than at 26 DPF (*P* < 0.01). There was a significant interaction effect between treatment and DPF on mRNA transcript abundance of *or 1-like* during the bloodworm transition at 50–80 DPF (natural-log transformed two-way ANOVA; F_2,35_ = 6.979, *P* < 0.01; Fig. 5i *or**1**-like*). *Post hoc* analysis revealed at 65 DPF the mRNA transcript abundance of L-alanine treatment was significantly higher than control (*P* < 0.01), but at 50 and 80 DPF there were no differences.

### Electro-olfactogram

We observed no significant main effect of treatment on amplitude of normalized EOG response in ~1-year-old lake sturgeon (natural-log transformed two-way ANOVA; F_2,71_ = 0.930, *P* = 0.338), although a significant main effect of cue on amplitude of normalized EOG response was observed (natural-log transformed two-way ANOVA; F_2,71_ = 61.061, *P* < 0.001; Fig. 6). *Post hoc* analysis determined the pellet cue had the greatest amplitude followed by 10^−3^ M L-alanine, then 10^−4^ M TCA.

## Discussion

We hypothesized that L-alanine exposure may influence the onset of feeding during dietary transitions from endogenous to exogenous feeding. We predicted that introduction of L-alanine during early life history would have a positive effect during dietary transitions and therefore promote growth and survival; however, the control treatment group was longer and weighed more than L-alanine treatment at 80 DPF; further, there was no difference in survival between the two treatment groups. Additionally, we hypothesized that olfaction in fishes is a developmentally plastic trait. We observed that acute treatment with L-alanine increased behavioural responses prior to the bloodworm transition and influenced all genes of interest throughout development, providing evidence of a developmentally plastic effect of L-alanine treatment with changes persisting till 50–65 DPF. Further, mRNA transcript abundance did not translate to changes in amplitudes of EOG responses, which would have been indicative of a long-term effect of L-alanine.

### Mortality and morphometrics

The increase in mortality seen in both groups after the completion of the bloodworm transition at 80–103 DPF is similar to the trends previously described in lake sturgeon (Yoon *et al.,* 2022) as once the smaller *artemia* are removed, sturgeon that failed to transition to the bloodworm diet perished. Similar trends in mortality during dietary transitions have been reported in other sturgeon species, too (Buddington and Christofferson, 1985; Gisbert and Williot, 1997; Boglione *et al.,* 1999; Gessner *et al.,* 2009). Furthermore, there was an interaction effect between treatment and DPF for body mass and length with the control treatment being heavier and longer than the L-alanine treatment at 80 DPF. The differences in body mass and length between treatments may be the result of the nature of a hatchery environment, i.e. being fed *ad libitum* three times a day to satiation. Although somewhat surprising, the proposed beneficial effects of introducing a food-related odourant (L-alanine) earlier in a hatchery environment seemingly had subtle negative effects on body mass and length. Indeed, some rationale for these differences may be drawn from the behavioural data.

### Behaviour

The only period where we show a difference in behavioural response was at 50 DPF where L-alanine-treated fish tended to move further than control. This result broadly supports two ideas; the first being a greater foraging effort during this period in L-alanine-treated fish, which was curiously at the beginning of the transitionary period from *artemia* to bloodworm and coincidental with the period of reduced growth in the L-alanine treatment. Increasing foraging effort in a resource-rich environment such as a hatchery would likely result in increased metabolic effort that would offset any advantage of increased feeding success (Garrido *et al.,* 2016). This would likely result in the differences in growth between L-alanine and control treatment groups as observed in the present study. Secondly, the change in behaviour could be related to a developmentally plastic response to L-alanine. However, we did not assess the behavioural response of lake sturgeon larvae to L-alanine. Similar increases in behavioural responses were observed following the exposure of lake sturgeon to the synthetic odours, phenethyl alcohol and morpholine (Kimmel *et al.,* 2023). In the Kimmel *et al.* (2023) study, fish were exposed at various developmental stages, but the most significant behavioural changes were observed in those exposed during the free embryo (12–19 DPF) and larval (19–49 DPF) stage, with exposure periods lasting 7–30 days. Interestingly, changes in behaviour were observed >50 days from exposure, whilst our observed behaviour change occurred at 50 DPF. In both cases, larval sturgeon were affected by short-term exposure to environmental odourants, suggesting some level of developmental plasticity within the olfactory system.

### mRNA transcript abundance

Differences in mRNA transcript abundance for all genes of interest were seen at 21 and 65 DPF. At 21 DPF prior to any addition of *artemia* there was a significant treatment effect for all genes, which opposed our prediction that L-alanine would only affect the mRNA transcript abundance of key genes associated with detection of dietary cues such as amino acids, specifically, the V2R and OR (Sato and Suzuki, 2001; Hansen *et al.,* 2003; Gerlach *et al.,* 2019). Although, there were initial differences at 17 DPF for the *taar 1-like*, which may have influenced the mRNA transcript abundance at 21 DPF. Nonetheless, the overall effect to the olfactory genes of interest could be related to environmental changes in comparison to diet (Yamamoto *et al.,* 2010), which could mean there were underlying downstream physiological effects of the L-alanine treatment that were not tested for in the present study.

The second time point that mRNA transcript abundance was consistently different across genes of interest was 65 DPF, approximately midway through the final dietary transition from *artemia* to bloodworm. The L-alanine treatment group throughout the bloodworm transition (50–80 DPF) had a consistent general increase in mRNA transcript abundance of genes of interest, although, with the exception of the *v2r 1-like* transcript, the increase was not significant. The timing of the increase in transcript abundance is supported by work with grass carp, *Ctenopharyngodon idella* (Pashchenko and Kasumyan, 1986), where olfactory sensory neurons were shown to increase severalfold once the lamellae folds appear and form the full olfactory rosette, which typically occurs prior to the bloodworm transition in other sturgeon species ~42 DPF (Boglione *et al.,* 1999; Kasumyan, 2011). Comparatively, control fish not exposed to L-alanine did not demonstrate consistent increases and generally mRNA transcript abundance was less at 65 DPF and significantly less for *or 1-like*, *v2r 26-like* and *v2r 1-like* compared to L-alanine-treated fish.

The relative decrease in mRNA transcript abundance of ciliated OSN related receptor genes *or 1-like* and *taar 1-like* throughout the artemia transition may in part be due to the lack of stimuli such as alarm cues and predator cues, which were not introduced in the present study. Studies have shown that lake sturgeon behaviour decreases in sensitivity to alarm cues as they develop from larvae into fingerlings in a hatchery setting (Wishingrad *et al.,* 2014) and Atlantic sturgeon larvae *Acipenser oxyrinchus* were predated on more frequently when raised in hatchery environment compared to other susceptible prey (Duda and Kapusta, 2019). Consequently, the ‘hatchery’ olfactory phenotype of reduced transcript abundance of key olfactory genes may be directly related to reduced performance of individuals to detect and appropriately respond to presence of alarm or predatory cues once released into the wild. Although, the testing of this was outside the scope and feasibility of this study.

### Electro-olfactogram

The observed differences in behavioural responses and mRNA transcript abundance prompted us to further investigate the potential long-term developmentally plastic effect of L-alanine exposure on lake sturgeon using EOG at ~1 year past exposure. In fishes, long-term olfactory developmental plasticity is generally termed imprinting (Harden *et al.,* 2006; Gerlach *et al.,* 2019; Gerlach and Wullimann, 2021). Specifically, experiments in salmonids have shown imprinting of single amino acids when exposed for 14 days during the parr-smolt phase (Yamamoto *et al.,* 2010). Importantly, the amino acids chosen were those dominating their natal stream and as such related to homing mechanisms related to spawning, which is not the case in our study. It has also been suggested that salmonids’ sensitive period is related to increases in thyroid hormone levels (Hasler *et al.,* 1983). Similarly, the critical window during which sturgeon may possibly become imprinted may include changes in whole-body thyroid hormone concentration and is suggested to take place when sturgeon are transitioning from endogenous to exogenous feeding (Boiko and Grigor’yan, 2002; Weinrauch *et al.,* 2023). We do not report any effect of L-alanine exposure from 17 to 20 DPF (prior to exogenous feeding) on EOG responses of 1-year-old lake sturgeon. However, we propose that a longer exposure period to L-alanine of at least 7 days may lead to observable changes.

## Conclusion

The current study provides evidence that odourants in the environment could play a role in early development of the olfactory system. Our prediction that the L-alanine-treated fish would have greater levels of growth and survival was not supported, but our behavioural metrics and mRNA transcript abundance of key olfactory genes were influenced by L-alanine treatment. Our behavioural results may be broadly supported by two explanations; the first being increased foraging effort, but any differences in growth were conceivably lost due to the abundance of food and the metabolic cost of increased foraging effort compared to the control group (Garrido *et al.,* 2016). The second being short-term phenotypic responses following exposure of L-alanine, with effects only lasting 50–65 DPF similar to the results found by (Kimmel *et al.,* 2023). Additionally, mRNA transcript abundance of key olfactory genes further supports the notion that exposure to odourants at key early life stage may have transient effects related to developmental plasticity of the olfactory system. The literature consistently demonstrates that low-performance phenotypes are produced in hatchery settings resulting in differences in foraging, feeding, homing and predator naivety in comparison to wild fish (Álvarez and Nicieza, 2003; Orlov *et al.,* 2006; Blanchet *et al.,* 2008; Roberts *et al.,* 2011; Saikkonen *et al.,* 2011; Stringwell *et al.,* 2014; Duda and Kapusta, 2019). Our findings demonstrate that introducing odourants during early developmental stages in lake sturgeon had phenotypic effects on the olfactory-related genes and behaviour; however, whether these changes would translate to low-performance phenotypes in the wild is unknown. Nevertheless, identifying essential odourants present in the wild and incorporating them into hatchery environments may help prepare individuals for better survival after release, contributing to the ongoing conservation effort of this imperilled species.

## Supplementary Material

Web_Material_coae084

## Data Availability

Data available on Mendeley data. Edwards, Tyler (2024), ‘Effect of L-alanine exposure during early life stage on olfactory development, growth and survival in age-0 lake sturgeon Acipenser fulvescens’, Mendeley Data, V1, doi: 10.17632/pn43r3f7nd.1
