# Correction to: Effect of L-alanine exposure during early life stage on olfactory development, growth and survival in age-0 lake sturgeon

**DOI:** 10.1093/conphys/coaf006

**Published:** 2025-01-18

This is a correction to: re Tyler Edwards, Ian A Bouyoucos, Caleb T Hasler, Mark Fry, W Gary Anderson, Effect of L-alanine exposure during early life stage on olfactory development, growth and survival in age-0 lake sturgeon *Acipenser fulvescens, Conservation Physiology*, Volume 12, Issue 1, 2024, 10.1093/conphys/coae084

In the originally published version of the manuscript, an incorrect image was published as Figure 5.

Figure 5 should read: 

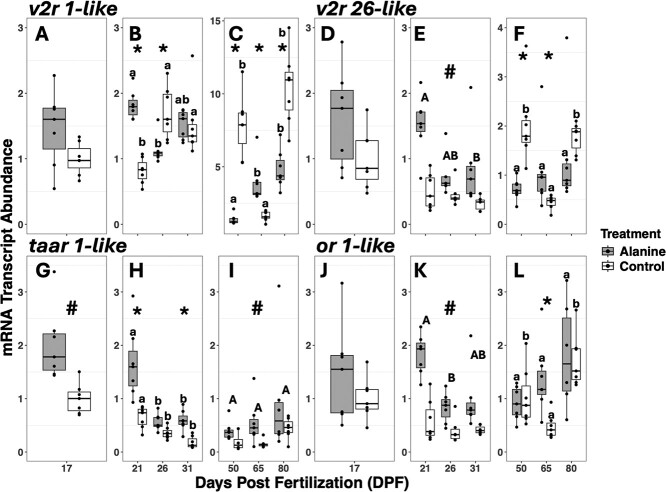



instead of: 

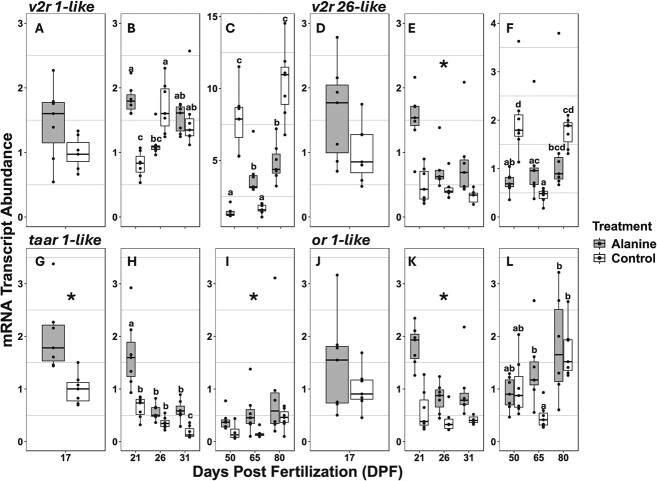



The emendation has been made to the article.

