# Supplementary material for: Effect of L-alanine exposure during early life stage on olfactory development, growth and survival in age-0 lake sturgeon Acipenser fulvescens
Source: Conserv Physiol. 2024 Dec 17;12(1):coae084. doi: 10.1093/conphys/coae084 (PMC11653895; doi:10.1093/conphys/coae084)
Supplement: Web_Material_coae084 [file web_material_coae084.docx]

Behavioural videos can be found at the link below:

Edwards, Tyler (2024). Conservation Physiology - Behavioural Videos. figshare. Media. https://doi.org/10.6084/m9.figshare.27849591.v1
